# Supplementary figures and images for: Redefining enteroaggregative Escherichia coli (EAEC): Genomic characterization of epidemiological EAEC strains
Source: PLoS Negl Trop Dis. 2020 Sep 8;14(9):e0008613. doi: 10.1371/journal.pntd.0008613 (PMC7500659; doi:10.1371/journal.pntd.0008613)

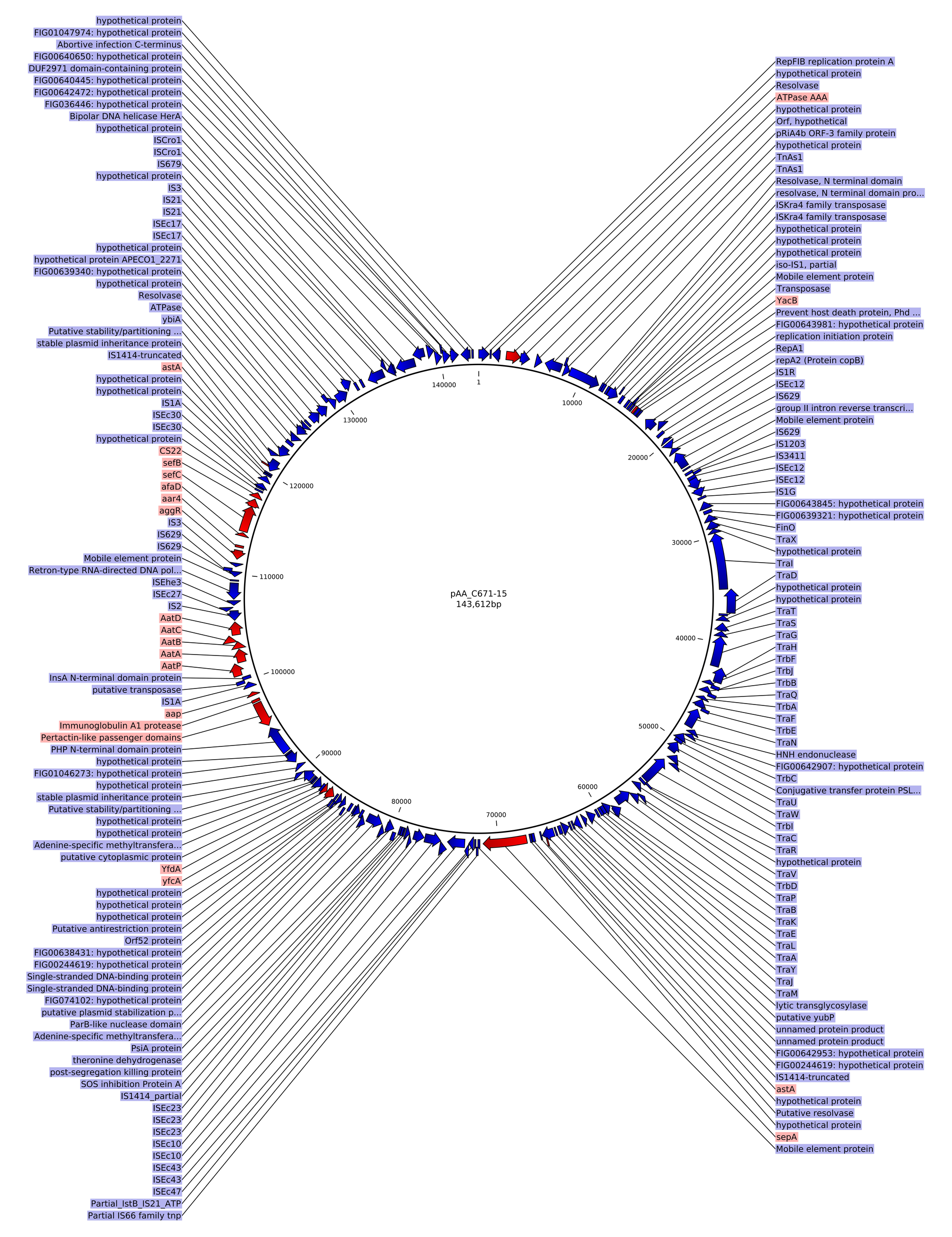

Supplement: S1 Fig — The plasmid was annotated using Rast as well as manual BLAST searches. Known virulence and putative virulence genes are shown in red. Other genes and open reading frames are shown in blue. (TIF) [file pntd.0008613.s001.tif]
