# Supplementary material for: Redefining enteroaggregative Escherichia coli (EAEC): Genomic characterization of epidemiological EAEC strains
Source: PLoS Negl Trop Dis. 2020 Sep 8;14(9):e0008613. doi: 10.1371/journal.pntd.0008613 (PMC7500659; doi:10.1371/journal.pntd.0008613)
Supplement: Attachment — Submitted filename: Letter to Reviewers_16072020.docx [file pntd.0008613.s006.docx]

**We thank the reviewers for their insightful and constructive suggestions. Please find below our responses to all review comments, as well as a description of the changes we have made in the manuscript per the recommendation of the reviewers.**

Reviewer #2: The objectives and the end outcome of the study needs to be better articulated. The authors have substantial work done but the objective for each section needs to be stated clearly.

**Thank you for this feedback, because it is important that the reader understands why we have done this study. We have added stated objectives in various parts of the manuscript. See manuscript**

Reviewer #1: The results are clearly and transparently presented and weaknesses appropriately identified.

The supplementary tables did not convert well into PDF, and as a result, the final PDF file has what appears to be 100s of pages of empty space.
**Noted**

Reviewer #2: Explain and define what is a control strain and how is it different from case strain?

**We have added the following. Line 84: Briefly, The GEMS is a 3-year, prospective, age-stratified, matched case-control study of moderate-to-severe diarrhoea in children aged 0–59 months residing in censused populations at four sites in Africa and three in Asia. Children with moderate-to-severe diarrhoea seeking care at health centers along with one to three randomly selected matched community control children without diarrhea were recruited. From patients with moderate-to-severe diarrhoea and controls, both clinical and epidemiological data were obtained, as well as anthropometric measurements, and a faecal sample to identify enteropathogens at enrolment.**

The results can be divided into sections and subsections each explaining a group of idea. Introduce in a line or two why what is done in the results sections so that it is each to follow the process.

**Noted. A sentence has been introduced to sections. See manuscript.**

Table 1: The legend needs to describe the method used and what is been shown in the table.

**Noted and added:** **“Prevalence and Distribution of Putative Virulence Genes in 97 EAEC Isolates from Cases and Controls by Whole Genome Sequence”**

Table 1: In the text please describe the odds ratio and why is it imp.

**Noted and added. Line 358: “EAEC, in general, was not associated with MSD in the GEMS study. To profile the virulence genes of our strain set, we blasted our 97 EAEC genomes for ~200 virulence and putative virulence genes found in DEC and ExPEC. As previously reported we found our EAEC strains to be highly diverse. Therefore, we quantified the strength of the association between cases and control calculating the odds ratio (OR).”**

Reviewer #3:

The results of the assay of adherence of colonoid monolayers are not properly clear.

**Duly noted: We have now better clarified this in the ms. Line 269. “Importantly, the CS22-like strains were negative for adherence to HEp-2 cells (see above). To ascertain if these strains were able to adhere to the human intestinal epithelium, we incubated CS22-like archetype strain, C671-15, with human colonic organoids and compared their ability to adhere with that of EAEC strain 042. As shown in Fig. 3, adherence between the two strains was nearly equivalent, and both adhered significantly more than an *aafA* null mutant”**

Reviewer #1: The conclusions are well supported, although the recommendation for reclassification of EAEC based on this paper may require further supportive data.

**Noted. We have rewritten the conclusion. Line 21 “Our data suggest that a molecular definition of EAEC could comprise *E. coli* strains harboring AggR and a complete AAF (I-V) or CS22 gene cluster. Further, it is possible that strains meeting this definition could be both enteric bacteria and urinary/systemic pathogens”**

Reviewer #1: In figure 4, the color shading of the phylogenetic groups themselves is not explained in the legend. I am assuming the colors were put in just to provide visual clarity about the separation of the groups, but given that the rest of the color schemes were clearly described, this became a bit confusing.

**Noted and added: “as well as colored sections within the figure (Phylogroup A – aqua; Phylogroup B1 yellow; Phylogroup D is separated into three sub-clusters of EAEC isolates (grey, blue and violet), with only the blue sub-cluster containing reference isolates”**

In Figure 3, the statistics should be shown on the figure itself.

**Noted. We have added statistics to Figure 3.**

Reviewer #1: This manuscript by Dr. Nadia Boisen et al describes a thorough genomic characterization of E. coli isolates characterized as EAEC based on molecular criteria from the MAL-ED study. This is an important work in the field, as heterogeneity of EAEC has complicated clinical studies for years. The most interesting finding is that of a previously unrecognized cluster of EAEC-like organisms that express the CS22 colonization factor associated with ETEC instead of AAF adhesins. The authors propose that the current molecular characterization of EAEC be expanded to include these strains, despite their lack of aggregative adherence on HEp-2 cells, based in part on their identification in children with diarrhea, and their adherence to human colonoids in vitro.

**As above, it is important that the reader understand why we think this manuscript is important, and we have added much more clarifying text. We think that this substantially improves the manuscript in fact. For the editor’s benefit, please note that in addition to the population structure of EAEC and the prevalence of virulence factors, we believe that fundamental contributions of the ms are 1) there is no evidence for additional AAFs beyond AAF/V (this will have major impact on vaccine development strategies); 2) strains without an AAF generally do not possess an otherwise complete AggR regulon; and 3) strains with CS22 (and only CS22) but not AAF may actually contain a complete AggR regulon, so CS22 may substitute for AAF. We have clarified these points in the text.**

The work as a whole is very thorough and transparently presented. The proposed reclassification is likely to engender good discussion among experts in the field, and might not be fully justified based on this current study, without further characterization in vitro and further work to associate these CS22 strains with disease (which can likely be done in the future in retrospective studies using isolates and clinical data from other longitudinal cohorts).

To improve the manuscript, I would suggest the following revisions:

1.In tables 1 and S4, there does not appear to have been a multiple comparisons correction used. I would recommend a statistical review to help determine whether this is needed in this case.

**This is an important point. Table S4: LS-BSR contains ~23000 loci, rendering any multiple correction analysis unfeasible. Multiple correction analyses are not customarily applied to this kind of analysis.**

**Table 1: After applying the Bonferroni correction or Benyamin-Hochberg (BH) procedure neither *sepA*, *iss*, nor *ompT* is significant. For this reason, validation is needed in further studies. It is notable (and we have noted it) that SepA has been previously associated with EAEC diarrhea.**

2.Table S4 lacks labeling of the columns to make the interpretation clear.

**Noted and changed**

3.The “negative” Hep2 assay (lines 197-199) does not describe whether these isolates demonstrated any mannose-resistant adherence at all, or just not an aggregative pattern. Based on the discussion later on, it appears that there was no adherence at all, but this should be stated in the results section

**Noted and added. Line 226:** **“None of the strains exhibited the AA pattern. Of all the strains assayed only three strains adhered to the cells but not with an AA pattern and one strain, C682-15, was cytotoxic”**

4. In the colonoid assays, I would like to know how other WT strains (e.g. HS and other nonpathogenic commensals) adhere in comparison to the three isolates shown in figure 3; i.e. is this specific to pathogenic strains? If not, then colonoid adherence may not be enough to put CD22 strains into the same pathogenic class as Hep-2-adherent strains. I’m also a bit surprised the authors didn’t cite the 2018 mBio paper (PMID: 29463660) that showed donor- and segment-specific differences in adherence to intestinal organoids.

**Noted. We have cited the mBio paper (PMID: 29463660). Also, we have changed the sentence (Line 272) to “As shown in Fig. 3, adherence between the two stains was nearly equivalent, and both adhered significantly more than an *aafA* null mutant [44] that exhibits adherence defects in this model system”**

**We have not done a comprehensive look at commensal *E. coli*. The important point of the experiment is that although CS22 strains do not adhere to HEp-2 cells, they nevertheless adhere well to the intestinal mucosa, weakening the pathogenetic relevance of the classical HEp-2 assay, and strengthening the argument that it is time to retire this phenotypic assay that dates to the 1970s.**

Reviewer #2: A general schematic or figure summarizing the goal/objective and workflow would help in better comprehension of the paper.

**Noted. As recommended by the reviewers we have adding explanatory sentences to Result sections and a clearer objective; we believe that these changes have greatly improved the manuscript.**
